# The antegrade continence enema procedure and total anorectal reconstruction

**DOI:** 10.1093/gastro/gou008

**Published:** 2014-03-12

**Authors:** Andrew P. Zbar

**Affiliations:** ^1^Department of Surgery and Transplantation, Chaim Sheba Medical Center, Tel Aviv University, Israel and ^2^Assia Medical Colorectal Group

**Keywords:** anal incontinence, antegrade continence enema, malone procedure, total anorectal reconstruction

## Abstract

Patients may present with anal incontinence (AI) following repair of a congenital anorectal anomaly years previously, or require total anorectal reconstruction (TAR) following radical rectal extirpation, most commonly for rectal cancer. Others may require removal of their colostomy following sphincter excision for Fournier's gangrene, or in cases of severe perineal trauma. Most of the data pertaining to antegrade continence enema (the ACE or Malone procedure) comes from the pediatric literature in the management of children with AI, but also with supervening chronic constipation, where the quality of life and compliance with this technique appears superior to retrograde colonic washouts. Total anorectal reconstruction requires an anatomical or physical supplement to the performance of a perineal colostomy, which may include an extrinsic muscle interposition (which may or may not be ‘dynamized'), construction of a neorectal reservoir, implantation of an incremental artificial bowel sphincter or creation of a terminal, smooth-muscle neosphincter. The advantages and disadvantages of these techniques and their outcome are presented here.

## INTRODUCTION

Anal (fecal) incontinence (AI) is characterized by uncontrollable episodes of an involuntary loss of stool at inappropriate times and in socially unacceptable circumstances [1, 2]. Although the incidence varies worldwide, there is a standard reported prevalence of AI in between 4% and 7% of the general population, with a higher estimate (up to 20%) recorded in patients who reside in nursing homes [3, 4]. Evidence would suggest that this symptom seriously impacts on patient-reported standardized quality of life and many aspects of healthy existence where—frequently because of embarrassment—most patients fail to seek specific medical help [5–7]. As a result of these decisions, there is a significant national, annual economic cost of conservative (i.e. non-surgical) care of these patients [8], part of which is influenced by the effect AI has on elderly patient institutionalization [9], as well as the inherent additional costs of anti-diarrheal drugs, healthcare visits, intermittent hospitalizations and patient payment for protective materials and pads. The additive costs of surgical therapies are significant and are impacted by their long-term success rates, the economic impact of procedure-related complications (which are considerable with some of the newer therapies) and the incidence of revisional operative procedures [10]. This article assesses the use and clinical results of antegrade continence enemas, either alone or in combination with total anorectal reconstruction following complete rectal extirpation, as valid surgical alternatives in the management of selected cases of AI.

## THE ANTEGRADE CONTINENCE ENEMA OR ‘MALONE' PROCEDURE

**Figure 1.**
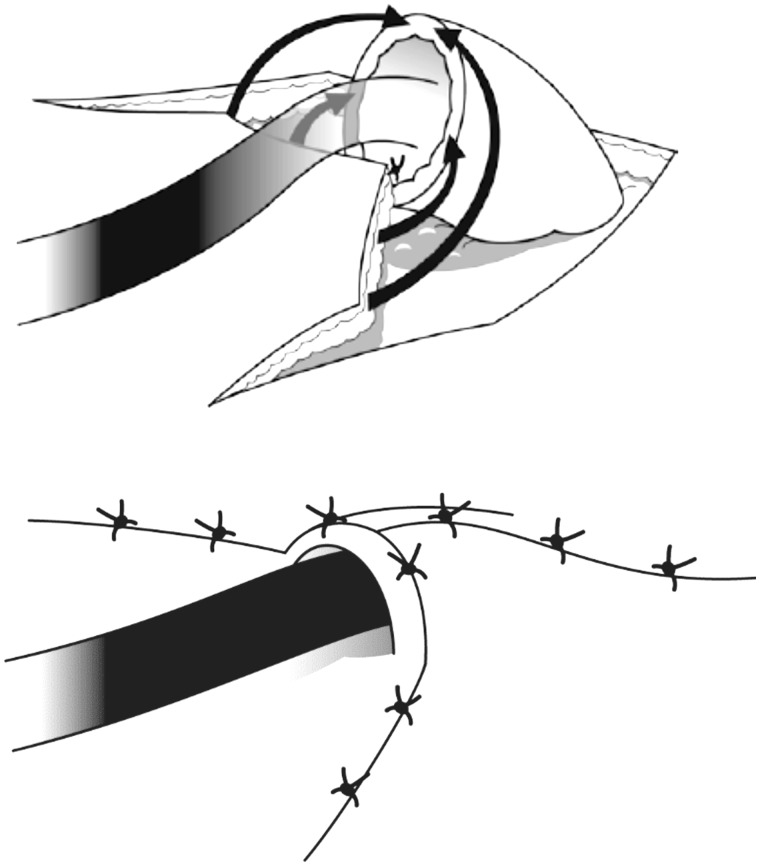
V–Y ACE procedure. The skin flap is sutured to the wall of either the appendix or a fashioned ileal conduit, with formation of a skin tunnel which covers the stoma. (*Reprinted with permission from Christensen P, Laurberg S. The Malone procedure and its variants. In Reconstructive Surgery of the Rectum, Anus and Perineum AP Zbar, RD Madoff and SD Wexner Eds. Springer 2013:273–282*).

**Figure 2.**
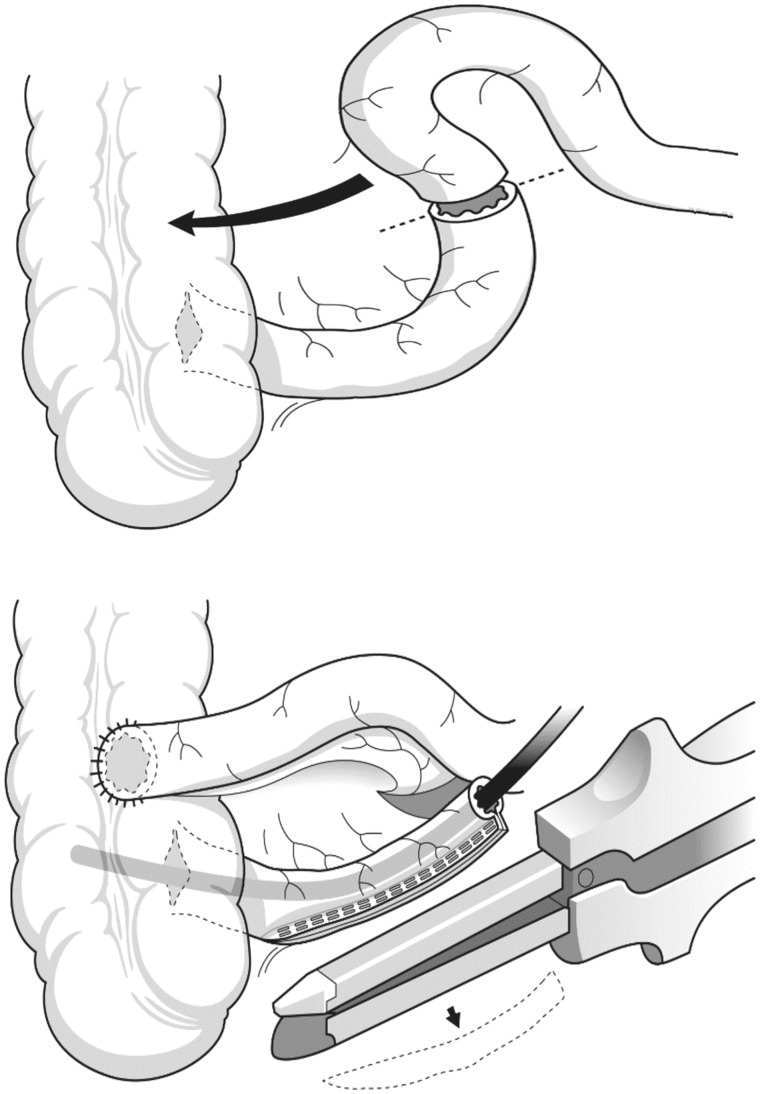
An ileocolic anastomosis is fashioned from the proximal ileum and the ascending colon with production of a small-caliber orifice for the stoma preserving the ileo-cecal valve. (*Reprinted with permission from Christensen P, Laurberg S. The Malone procedure and its variants. In Reconstructive Surgery of the Rectum, Anus and Perineum AP Zbar, RD Madoff and SD Wexner Eds. Springer 2013:273–282*).

An alternative for the management of various cases of AI that have resisted other more conventional forms of treatment is the operative technique of antegrade continence enema (ACE), rediscovered by Malone [11]. This approach is more popular in Europe, where it was originally reported in 1905 [12]. The basic technique was adapted from the Mitrofanoff procedure used for a continent catheterizable stoma leading to the bladder [13], but using an appendicostomy for antegrade colonic irrigation. Originally, it was used as a cleansing treatment in *spina bifida* patients presenting with incontinence. The original operative description used the appendix as a continent, catheterizable, abdominal stoma, which was reversed and placed in a submucosal tunnel of the cecum to form a non-refluxing channel. This was modified to a simpler, non-reversed design with or without creation of a definitive anti-reflux mechanism [14]. The Malone procedure may be carried out on the right iliac fossa, employing a V–Y cutaneoplasty with intermittent catheterization, using a Foley's catheter for creation of a continent, usable conduit under the skin (Figure 1). In the event that there has been a prior appendectomy, or where the appendix is atrophic, the cecal wall can be used as a flap, or a flap may be constructed from the terminal ileum, with the latter being the preferred method overall; this is performed by transecting the ileum about 15 cm from the ileocecal valve and turning the vascularized segment outwards as a buried stoma with neo-ileocecal anastomosis (Figure 2) [15–18]. Latterly, part of this procedure may have been laparoscopically assisted [19].

Usually after a two-week waiting period—which allows the system to heal and mature—enemas are then progressively increased in volume up to 1 L, with the final regimen determined by trial and error ,as well as by patient tolerance. Results, in both children and adults who are motivated, appear to be acceptable in both the short- and the long terms [20–40]. Long-term quality-of-life data is sparse, where it has been shown that motivation and usage diminishes over time [41]. The results overall appear better in those with neurogenic bowel disability [30, 36]. The principal morbidity of the procedure includes stomal complications, such as stenosis in between one-quarter and one-half of cases during follow-up, and stomal irritation due to refluxing mucus discharge [42]. The complication rate is high but often relatively minor in nature, with fewer problems if the tapered neo-ileal conduit design is used [43]. 

Latterly the ACE procedure has been performed as a percutaneous, endoscopic colostomy which was originally used in the treatment of intermittent sigmoid volvulus [44, 45]. The comparative functional results appear excellent, although there is a considerable morbidity which, in a small percentage, can be life-threatening [46, 47]. Norman Williams and his group from the London Hospital have used an alternative here, describing a continent colonic conduit with a full-thickness intussuscepted valve, similar to a Kock continent ileostomy [48], with others describing a retubularized ileal segment for this purpose [49]; still others using a retubularized stomach segment [50]. It would appear that antegrade irrigation provides better results than retrograde irrigation [26, 51, 52], although patients should be warned that some symptoms such as bloating—and nausea if there is coincident constipation—may be essentially unaffected. 

A range of fluids may be used for irrigation purposes, including phosphate solution, tap water, saline, phosphosoda, polyethylene glycol, liquorice root solution or arachis oil. Caution is advised in small children and fragile, elderly patients, as well as in those with chronic renal failure [53, 54]. Table 1 shows the reported outcomes of ACE-related procedures in a range of disorders that were combined with primary AI.

**Table 1.** Antegrade continence enema-related reported outcomes

Author [Ref]	Indication	Number	Success	Complications
Hill [20]	Slow transit	6	6	50%
Christensen [26]	Neurogenic	8	7	38%
Rongen [27]	Slow transit	12	8	83%
Teichman [30]	Neurogenic	6	5	67%
Lees [31]	Slow transit	32	15	88%
Hirst [32]	Obstructed defecation syndrome	20	13	85%
Portier [33]	Mixed	28	28	50%
Lefevre [35]	Mixed	22	18	20%
Poirier [36]	Mixed	18	14	56%
Altomare [37]	Mixed	11	8	–
Koivusalo [38]	Mixed	27	24	63%
Worsoe [40]	Mixed	69	51	38%

ODS = obstructed defecation syndrome

## TOTAL ANORECTAL RECONSTRUCTION

Total anorectal reconstruction (TAR) is a method of neorectal reconstruction following complete rectal and sphincter excision. The concept was first proffered in 1930 by Chittenden, who performed a continent perineal colostomy using a flap of the *gluteus maximus* as a neosphincter [55], with Margottini reporting a large series of this approach in 1950 [56]. The coincident surgical developments of muscle transfer procedures, techniques of dynamization through electrical field stimulation, artificial implants and myogenic sphincter augmentation techniques have been applied to this approach in the development of TAR. The design makes no real attempt to restore those normal functions that are lost, including an adaptable neorectal reservoir, capability of storage and intermittent discharge, a complex closure (sphincteric) mechanism and a discriminatory sensory apparatus, the arms of which are part of normal continence and, as such, full continence cannot be guaranteed for patients undergoing a TAR. 

TAR has been made technically feasible in selected cases by the creation of a neorectal reservoir, along with supplementation using autologous muscle or an artificial sphincter. An additional supplement would be the use of an appendicostomy (or an ileal/colonic conduit) for antegrade (ACE) irrigation, as described above, with the result of a ‘pseudo-continent' status in the patient [57]. Substitution for the rectal functions of storage and sensibility can further be achieved with a segment of descending colon, which has a propulsive function and limited storage capacity, although there is extensive evidence to show that many patients (at least 50%) have a significant ‘low anterior resection syndrome' after low restorative proctectomy, characterized by an increase in the number of daily bowel motions, nocturnal urgency, stool fragmentation, irregular/incomplete defecation and even frank tenesmus and incontinence [58]. In those who have undergone total rectal excision, the lack of sensory receptors in a peri-anal colostomy results in universal passive incontinence whereas, in those with some area of rectal sensation remaining, data from the creation of various forms of neorectal reservoir (such as the colonic 'J' pouch, the side-to-end Baker anastomosis or the coloplasty) suggests that improvements in function over time are due to the reduced action of neorectal motor activity, rather than its role (and capacity) as a true reservoir [59].

In the specific circumstance of TAR, if a pouch is constructed, the shape of the neorectum needs to conform to the anatomical type of reconstruction, where the distal 3–4 cm of the colon will be surrounded by a neosphincter. For this purpose, ‘J' pouch construction has been combined with a gracilis neoanal sphincter in dogs [60], as well as in humans [61–63]. Geerdes *et al.* [64] described a pouch placed just proximal to a gracilis wrap, opening the colon anti-mesenterically over a length of 15 cm and covering the defect with an isolated patch segment of distal ileum. As an alternative, Williams *et al.* used a triplicated ileal pouch as 15 cm limbs, combined with a stimulated graciloplasty, for the same purpose [65]. Both of these complex techniques were not, however, associated with particularly good function. A simpler approach is to translate a 6–7 cm long coloplasty above a colo-anal anastomosis, as advocated by Fazio and colleagues [66], or by Devesa, who performed a longitudinal colonic myotomy proximal to a neosphincter, designed to diminish the peristaltic activity of the descending colonic segment [67].

The second approach is that of sphincter substitution, where it is increasingly understood that IAS damage leads to serious continence disturbance in some cases. The issue of IAS implantation and augmentation is discussed elsewhere in this special edition. In this respect, Torres *et al.* originally described a neo-internal anal sphincter [68], which was wrapped in a spiral configuration around a colonic pull-through similar to that described by others [69, 70]. In this technique, 3–4 cm of distal colon is freed from the pericolic fat and the seromuscular layer is dissected away from the mucosa, creating a smooth-muscle sleeve which is then incised in a spiral fashion. The effect is to construct a pedunculated muscular flap, 1.0–1.5 cm wide and 5–7 cm in length, which is then wrapped around the bowel and fixed to its wall. This creates a cone-shaped smooth muscle cuff attached to the terminal part of the colon. Results of this technique have been variably reported [71–75], with Lorenzi *et al.* modifying this approach by denuding the mucosa and then everting the last 1.5 cm of the colonic end, which is then anastomosed to the neo-anus in the perineal skin [76]. Physiological studies have shown that these areas distally develop a high pressure zone and a passage pressure gradient. The role of this added approach is unclear, where free grafts obviously lack intrinsic and extrinsic innervation and where they may function more as a biological peri-anastomotic sling and as a barrier to evacuation, than as a true functional neosphincter.

A variety of muscles have been used as translation, for the management of AI, to those patients undergoing TAR, including the *gluteus maximus*, the adductor musculature and the gracilis.

This technique has been supplemented by Farid of Egypt with *fascia lata* in very specialized AI patients after reconstruction of congenital anorectal anomaly [77], although the use of a gluteoplasty in adult TAR data is limited [78]. Yuri Shelygin's Moscow group has described success in 82% of patients treated with an *adductor longus* reconstruction TAR in the only report available [79]. Jacob and colleagues first used a static (adynamic) graciloplasty for the purposes of TAR for a congenital anomaly [80], with Simonsen *et al.* using the technique after rectal cancer excision [81]. The data here are limited [82–86]; however, the largest series of dynamic graciloplasties for TAR reported by Cavina *et al.* showed an 87% success rate in 98 patients after 55 months of follow-up, although there was significant morbidity in one-third of cases [83]. The dreaded complication is necrosis of the neo-anus, which appears to occur particularly in the TAR cases [87].

Another approach, by Romano *et al.*, is formal sphincter reinforcement with an artificial anal sphincter with translation to those specialized patients after abdomino-perineal excision [88]. The initially good results seen in his eight cases prompted similar work by Devesa *et al.* in a small number of cases, but the high rate of complications and the need for explants (as in those patients treated primarily for AI) did not result in extensive use of this technique [67]. The use of an anal sling as a supplement to TAR (a subject covered elsewhere for the management of AI in this special edition) has not been reported.

Others have reported the use of an antegrade continence enema technique for specific use in TAR cases. Chiotasso *et al.* first reported its use in conjunction with a perineal colostomy [89], where Farroni and colleagues compared the quality-of-life parameters of those with a perineal colostomy and an appendicostomy with those with an abdominal colostomy, concluding that the perineal colostomy with appendicostomy for was a viable option [90]. As per the standard ACE procedure, if the appendix is not available, an ileal neo-appendicostomy, cecal flap or colonic conduit may be fashioned. The advantage of providing ‘pseudo-continence' in these patients is the secondary avoidance of fecal impaction, which can be a very disabling symptom after TAR, particularly where an external sphincter recreation or substitution has also been performed.

Much of the available literature in this specialist group of patients is difficult to interpret, where congenital anomalies that have been reconstructed are mixed with cases where radical rectal extirpation for cancer has been carried out, and where the procedures performed are heterogeneous and combined. Apart from comparing quality-of-life parameters, another way of expressing satisfaction with the procedure might be the comparison of patients' quality of life scores between those with an abdominal stoma and those in whom there is reconversion to a perineal stoma [91]. Such an approach requires a revision of the way in which we assess quality of life in incontinent patients following reconstructive surgery.

Table 2 shows the outcomes of dynamic and adynamic graciloplasty alone for TAR. In this group there is a high morbidity and surgical revision rate, with normal continence reported in only 20% of evaluable patients. At least one year is required to achieve acceptable continence in these cases. There does not appear to be any advantage in ‘dynamizing' the graciloplasty in some series [81, 84, 92], suggesting that the functional results of graciloplasty would be more attributable to the biological ‘cerclage' effect with the gracilis, rather than to the stimulation itself. If this is true, then most of the perineal stomas treated by explantation of the stimulator would have either undergone re-implantation or been reconverted to an abdominal stoma. Further, severe constipation after graciloplasty has almost always been a feature of stimulated cases [93].

**Table 2.** Dynamic and adynamic graciloplasty as a supplement to total anorectal reconstruction

Author [Ref]	Number	Dynamic/ adynamic	Complications	Function
Santoro [92]	14	0/14	1 converted	73% pseudocontinuous
Mander [61]	10	10/0	80%	All wore pads
Geerdes [64]	16	16/0	4 reconverted	30% continent
Cavina [83]	98	98/0	37%	87% continent
Rullier [84]	15	0/15	73%	78% continent
Ho [86]	17	17/0	40%	45% continent
Simonsen [81]	24	0/24	65%	77% continent
Violi [85]	23	15/8	37%	75% continent
				87% dynamic
				38% adynamic

Table 3 shows the outcomes of perineal colostomy with a colonic smooth-muscle wrap and colonic irrigations as part of TAR. As patients do better with colonic irrigation, the value of a neosphincter remains somewhat questionable. Table 4 shows the outcomes if an artificial implanted sphincter device is used in TAR, and Table 5 shows the functional outcome if TAR incorporates an ACE procedure in the management. In this latter group, ileal/cecal/colonic conduit procedures are technically more complex and carry a higher morbidity rate than a simple appendicostomy. Late complications are usually related to stomal stenosis, which can be easily managed by a temporary catheter at night or by a surgical V–Y plasty. Stomal leakage and reflux may be prevented by a cecal imbrication—somewhat akin to a Nissen fundoplication [94]. This approach particularly appears very viable for young patients with AI and prior congenital anorectal anomalies [95]. Overall, the ACE procedure contributes to the avoidance of constipation after TAR when external sphincter reconstruction or substitution has been performed, and where it would appear that in all procedures in which ACE was associated, the good functional results are due to colonic irrigation rather than the other more complex aspects of the technique.

**Table 3.** Data pertaining to smooth muscle neosphincters combined with colonic irrigation for total anorectal reconstruction

Author [Ref]	Number	Complications	Functional status
Lasser [73]	40	55%	11% continent 5% reconverted
Gamagami [74]	63	65%	39% satisfactory
Portier [75]	18	33%	No reconversions
Pocard [96]	12	Not stated	92% pad use
Hirche [97]	44	40%	50% continent

**Table 4.** Artificial bowel sphincter use in total anorectal reconstruction

Author [Ref]	Number	Complications	Functional result
Romano [88]	8	–	87% continent
Lirici [98]	3	All explanted	All continent
Devesa [67]	1	Explanted	Improved
Ocares [99]	1	Explanted	Not evaluable

**Table 5.** Combined procedures for total anorectal reconstruction with antegrade colonic irrigation

Author [Ref]	Number	Complications	Functional status
Saunders [100]	14 Continent colonic conduit + stimulated graciloplasty	71%	50% continent
Farroni [90]	13 Malone cecal conduit	Not stated	85% continent
Ardelean [101]	9 Antegrade continence enema + posterior sagittal rectoplasty	Not reported	All continent and clean

In summary, the role for TAR (and its preferred technique) is currently unclear. In its use, patients and their families need to be informed that continence will effectively never be perfect. The two main candidate groups for this procedure include those with imperfect continence after surgery for a congenital anomaly as children or infants, and those who request reconstruction after radical rectal extirpation for cancer. Patients must understand the morbidity of any proposed procedure and the reported likelihood of subsequent revisional surgery over time. In cancer, after exclusion of recurrent disease, other factors such as obesity, intra-abdominal adhesions, comorbidity, prior perineal irradiation and even age may be precluding conditions for TAR. The issue of immediate TAR vs delayed TAR is also controversial and somewhat akin to the argument of immediate vs delayed breast reconstruction after mastectomy. It would seem feasible to perform a perineal colostomy and an appendicostomy for ACE at the initial rectal excision in motivated cases, and this may be associated with quite minimal perineal morbidity in early selected cases (those with T1-2N0 tumors) when compared with the known perineal morbidity of primary perineal closure.

### Conflict of interest: none declared.
